# Formulation of Metformin-Loaded Chitosan Nanoparticles and In Vivo Evaluation of Its Hypoglycemic Effects

**DOI:** 10.3390/molecules31091539

**Published:** 2026-05-06

**Authors:** Zainab Omeed Awchee, Airemwen Collins Ovenseri, Ahmad Saleh Malkawi, Leyla Beba Pozharani

**Affiliations:** 1 Faculty of Pharmacy, Cyprus International University, Nicosia 99258, Cyprus; acollins@ciu.edu.tr; 2 Department of Pharmaceutical Sciences, Faculty of Pharmacy, Jadara University, Irbid 21110, Jordan; malkawi_ahmad@live.com; 3 Department of Pharmaceutical Sciences, Faculty of Pharmacy, Institute of Graduate Studies and Research, Eastern Mediterranean University, Famagusta 99628, Cyprus; leyla.beba@emu.edu.tr

**Keywords:** chitosan nanoparticles, drug release kinetics, ionic gelation, metformin, sustained drug delivery

## Abstract

This study formulated and characterized metformin-loaded chitosan nanoparticles (NPs) using the ionic gelation technique and evaluated the drug release kinetics. Characterization confirmed successful drug encapsulation, with Fourier-transform infrared spectroscopy (FTIR) indicating compatibility, and X-ray diffraction (XRD) showing attenuation of characteristic metformin reflections consistent with reduced crystalline contribution after encapsulation. Particle sizes ranged from 74.28 to 86.82 nm. The NPs exhibited stable zeta potentials (+42.38 to +49.06 mV) and high entrapment efficiencies (68.42–81.26%). In vitro drug release studies at pH 7.4 and pH 2.0 demonstrated an initial burst release, followed by sustained release over 24 h. The cumulative drug release ranged from 81.92% to 97.72% at pH 7.4 and 89.4% to 98.1% at pH 2.0, with a faster release at pH 2.0. Drug release kinetics followed first-order for batch MN1, while batches MN2 and MN3 best fitted into the Higuchi model, indicating diffusion-controlled release through the chitosan polymeric network. The formulated metformin nanoparticles demonstrated significant potent dose-related and time-dependent cytotoxic effect against ovarian cancer cell lines and in vivo blood glucose lowering effect compared to the conventional dosage forms and control (*p* < 0.05). These findings highlight the potential of metformin-loaded chitosan NPs for sustained drug delivery, which may enhance patient compliance by reducing dosing frequency. Future studies should further explore their clinical applications.

## 1. Introduction

Nanopharmaceuticals are drug formulations prepared at the nanoscale for targeted or site-specific delivery to various organs in the body, either passively or actively [1]. They can improve bioavailability, enable controlled or sustained drug release, and enhance site-specific delivery owing to their small particle size and high surface-area-to-volume ratio. These systems have been investigated for applications in diagnosis, prophylaxis, and treatment and can be manufactured using synthetic or natural biodegradable polymers [2,3,4]. The growth of nanoscience and nanotechnology has also supported the development of nanomedicine and nano-enabled drug-delivery approaches [5].

Nanopharmaceuticals present new potential for drugs that were previously unsuitable for conventional oral or parenteral dosage forms. These drugs can be formulated as nanoparticles for site-specific delivery due to their improved pharmacodynamics/pharmacokinetics and intracellular delivery [3,6]. This approach can increase pharmacological action and decrease adverse drug effects, leading to enhanced therapeutic efficacy. Nanopharmaceuticals may also improve the biologic half-life of drugs by inhibiting their metabolism. Formulating drugs as nanopharmaceuticals is an ideal drug delivery strategy, particularly for drugs requiring improved stability, solubility, permeability, or controlled release [1,3,5].

Diabetes mellitus (DM) is a metabolic endocrine disorder characterized by hyperglycaemia and includes different forms, such as type I, type II, gestational, neonatal, and monogenic diabetes. Type I diabetes is strongly associated with autoimmune mechanisms and HLA-related susceptibility [7], while clinical studies of insulin-dependent diabetes have documented associated vascular complications such as hypertension [8]. Monogenic diabetes, including maturity-onset diabetes of the young (MODY), has distinct molecular mechanisms and remains underdiagnosed in clinical practice [9,10]. Type II diabetes is primarily associated with insulin resistance and relative insulin deficiency, and chitosan-based biomedical strategies have also been reviewed in relation to diabetes management [11].

Metformin is an oral anti-hyperglycaemic medication commonly used to treat type II diabetes mellitus; it is a biguanide that lowers both postprandial and basal plasma glucose levels by suppressing hepatic glucose production, decreasing intestinal glucose absorption, and enhancing glucose uptake and utilization [12]. Chitosan and its derivatives have been reviewed for potential antidiabetic mechanisms [13], while metformin has been incorporated into different nanocarrier and biguanide-based dosage-form systems for improved drug delivery [14,15]. Metformin is classified in the Biopharmaceutical Classification System (BCS) as a class III drug, meaning it is highly soluble, slowly permeable, and poorly absorbed. Due to its short half-life (1.0–4.5 h), poor bioavailability (40–60%), and rapid release from conventional tablet formulations, patients need to take the drug 2–3 times a day, necessitating repeated doses (1.5–2.0 g/day) or a 3-daily-dose regimen to maintain therapeutic plasma concentrations [12,16]. Diabetic patients often require chronic therapeutic treatment and management with multiple doses, which can lead to reduced adherence to medication and an increased risk of adverse effects [12,16,17]. Many researchers have been studying ways to increase the bioavailability of metformin to achieve its maximal therapeutic effect [18]. Chitosan–tripolyphosphate nanoparticles and chitosan-based delivery systems have been investigated for controlled delivery and diabetes-related therapy [17,19,20]. Formulating metformin nanoparticles (NPs) may help overcome these challenges by providing an initial dose followed by sustained release of the maintenance dose, ultimately increasing the bioavailability of metformin, reducing dosing frequency, and improving patient adherence to therapy [18,20]. Therefore, the aim of this research was to prepare metformin nanoparticles using the ionic gelation technique and to evaluate their morphology, crystallinity, entrapment efficiency, drug release kinetics, cytotoxic and in vivo hypoglycemic effect.

## 2. Materials and Methods

### 2.1. Materials

Metformin as a pure sample was obtained from Ranbaxy Laboratories Limited (Gurgaon, India), chitosan (86% acetylation) and streptozotocin were purchased from Sigma-Aldrich (Schnelldorf, Germany), tripolyphosphate (TPP) (Meron Biopolymer, Cochin, Kerala, India), ninhydrin was provided by Sisco Research Laboratories (Mumbai, India), and acetic acid was obtained from Cipla Limited (London, UK). The other chemicals were of analytical grade.

Adult male Wistar rats weighing between 200 and 250 g were purchased and kept at the Animal House Facility of the Faculty of Pharmacy, University of Benin, Nigeria. They were kept under standard conditions (12 h light/dark, 25 ± 0.1 °C), with ad libitum access to food and water and allowed to acclimatize for two (2) weeks before the experiments commenced. The Faculty of Pharmacy Ethics Committee granted the Ethical approval with Reference No.: EC/FP/018/25. All animal experiments were performed according to standard procedures previously reported by Alhajj et al. [6].

### 2.2. Methods

#### Preparation of Metformin NPs

**Figure 1:**
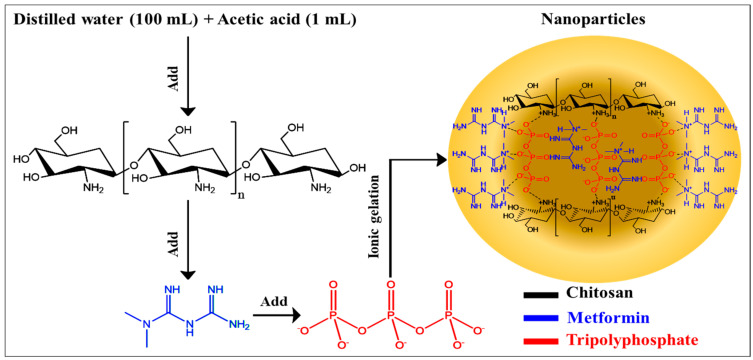
Illustration of the various steps followed for synthesizing metformin NPs.

The ionic gelation method was used to formulate the metformin nanoparticles (NPs) according to the procedure previously described by Salem [21]. Figure 1 presents a schematic illustration of the followed steps in synthesizing metformin NPs. Chitosan (50 mg) was weighed and dissolved in a mixture of distilled water (100 mL) and acetic acid (1 mL). Metformin (100 mg) was added to the solution. The mixture was continuously agitated using a magnetic stirrer (1500 rpm) for 1 h. A tripolyphosphate (TPP) solution was prepared by dissolving 20 mg of TPP in 10 mL of distilled water and was added dropwise to the metformin solution, followed by continuous stirring for 3 h. The preparation was then homogenized and centrifuged for 30 min at 3000 rpm. The formulated metformin NPs were lyophilized using a freeze dryer (Labstac LLC, Pittsfield, MA, USA) at a temperature of −25 °C and a pressure of 600 Pa and were then collected. Three different formulations were prepared by varying the amounts of metformin, TPP, and chitosan and were designated MN1, MN2, and MN3 (Table 1).

### 2.3. Evaluation of the Metformin NPs

#### 2.3.1. Fourier Transform Infra-Red (FTIR) Spectroscopy

The functional groups present in the pure metformin sample and the optimized metformin nanoparticles (NPs) were analyzed using an FTIR spectrophotometer (Shimadzu, Kyoto, Japan) with the potassium bromide (KBr) pellet technique. A sample (1 mg) was mixed with KBr (40 mg) and compressed into pellets using a manual press. The spectra were scanned over a range of 4000–500 cm^−1^. FTIR peak interpretation for metformin was compared with previously reported ATR-FTIR data [22].

#### 2.3.2. X-Ray Diffraction (XRD) Analysis

The crystallinity of the pure metformin sample and the optimized metformin nanoparticles (NPs) was analyzed using an X-ray diffractometer (XRD, Rigaku Rint 2000, Tokyo, Japan). Data was collected using a Cu anode and a monochromator voltage of 40 kV. Using a continuous scan, the diffraction pattern was recorded over the range of 2° < 2θ < 80°.

#### 2.3.3. Scanning Electron Microscopy

The surface morphology of the metformin nanoparticles (NPs) was analyzed using a scanning electron microscope (SEM) (Shimadzu, Kyoto, Japan). Samples were placed on a metal stub and vacuum-coated with gold before imaging.

#### 2.3.4. Particle Size, Zeta Potential, and Polydispersity Index (PDI) Analysis

The polydispersity index (PDI), zeta potential, and particle size of the metformin nanoparticles (NPs) were determined using a Zetasizer Nano ZS (Malvern Instruments, Malvern, UK). Measurements were performed at 25 °C after appropriate dilution with distilled water [21,23].

### 2.4. Drug Entrapment

The encapsulation efficiency study was conducted using the method previously described by Alhajj et al. [6] with some modifications. To the vessels containing the lyophilized metformin NPs, deionized water was added in 50 mL volumes and gently stirred manually for 3 min using a glass rod to dissolve excess metformin. The vessels subsequently were set free to allow settlement of the solids and obtain clear supernatants. The clear supernatants were used to quantify non-encapsulated metformin according to a previously reported metformin ninhydrin method [24]. Briefly, this method is based on the formation of ninhydrin conjugate with the metformin primary amino group, forming a spectrophotometrically detectable violet color under alkaline conditions. Therefore, aliquots of 0.5 mL were taken from all of the supernatants and added to 25 mL volumetric flasks containing 1.5 mL of 5 M NaOH, 2.2 mL of 1% ninhydrin solution, and 10 mL of water with 30 min heating on a water bath. The mixtures were cooled, and the final volumes in the flasks were adjusted to 25 mL by adding deionized water. Each flask was aliquoted to measure absorbance at a 570 nm wavelength using a UV spectrophotometer (Shimadzu, Kyoto, Japan). Following the method described above, metformin standard solutions of known concentrations were prepared to construct a calibration curve (absorbance vs. concentration), which was used to quantify metformin in all subsequent measurements. The entrapment efficiency (EE) was primarily determined using the indirect method by measuring the amount of free (unentrapped) metformin in the supernatant after nanoparticle separation. The concentration of metformin in the supernatant was calculated from the calibration curve, and EE was calculated using Equation (1):

(1) $$EE\;(\%)=\frac{Reference\;absorbance-Supernatant\;absorbance}{Reference\;absorbance}\times 100$$

Furthermore, to validate the indirect method, EE was also determined using a direct approach by quantifying the entrapped drug after complete digestion of the lyophilized metformin NPs. The concentration of metformin released from the digested nanoparticles was determined using the same calibration curve, and the total entrapped amount was calculated. Briefly, a known weight (10 mg) of the lyophilized metformin NPs was accurately weighed and re-dispersed in 1 mL of deionized water. To dissolve the chitosan–TPP NPs matrix and release the entrapped metformin, 1 mL of 1% *v*/*v* acetic acid was added to the dispersion. The mixture was stirred at room temperature for 1 h to ensure complete digestion of the nanoparticles. The resulting solution was centrifuged at 5000 rpm for 10 min to remove any undissolved excipients or debris. The clear supernatant was collected and analyzed spectrophotometrically at 570 nm using the ninhydrin assay, following the same procedure described above. The amount of metformin released from the digested nanoparticles was used to calculate the entrapment efficiency (EE) percentage using Equation (2). Moreover, the loading capacity (LC) percentage of metformin NPs thereafter was calculated using the amount of metformin entrapped and the total mass of metformin NPs recovered after lyophilization using Equation (3).

(2) $$EE\;(\%)=\frac{Entrapped\;drug\;in\;dissolved\;NPs}{Total\;drug\;used\;in\;formulation}\times 100$$

(3) $$LC\;(\%)=\frac{Entrapped\;drug\;in\;NPs\;(mg)}{Total\;weight\;of\;NPs\;(mg)}\times 100$$

### 2.5. In Vitro Metformin Release Studies

The in vitro drug release study was conducted using a Type II dissolution apparatus, following the method previously described by Bao et al. [25]. The drug release studies were performed in two media: (1) phosphate-buffered saline (PBS, pH 7.4) composed of disodium hydrogen phosphate, sodium dihydrogen phosphate, and sodium chloride; and (2) acidic medium at pH 2, prepared by adjusting a 10 mM phosphate buffer with 1 M HCl until the desired pH was reached. Colloidal dispersions of metformin nanoparticles (NPs) in release media at 2 mL volumes were placed in dialysis membranes (HiMedia, Mumbai, India) and immersed in release media either at pH 7.4 or pH 2.0 with a temperature maintained at 37 ± 0.5 °C. Sink conditions were maintained during the in vitro release study by using 100 mL of release medium, which is sufficient to fully dissolve the maximum possible amount of metformin released from the nanoparticles. The aqueous solubility of metformin, which shows freely soluble drug molecules, ensured that the medium remained unsaturated throughout the experiment. The release media were continuously stirred at 200 rpm, and 1 mL samples were withdrawn at predetermined intervals using a pipette and analyzed with a UV spectrophotometer at 232 nm. An equal volume of fresh release media was added to the release apparatus to maintain proper sink conditions. As a control, an amount of pure metformin powder equivalent to the drug content in the metformin NPs was dissolved in the release media and tested under the same conditions as the formulations. An additional control was prepared by digesting a known amount of lyophilized metformin-loaded NPs (equivalent to the amount of entrapped drug) using 1% *v*/*v* acetic acid, as described above. The resulting solution, containing the fully released metformin, was added to the release media. This control was used as a reference to compare drug release from intact metformin NPs at each time point.

### 2.6. Determination of the Released Metformin

At the end of drug release studies, the maximum concentration of the released metformin from the loading NPs was determined using the ninhydrin-based method described above [24]. Sufficient aliquots of the released metformin were collected and separately placed in reaction vessels, each containing 1.5 mL of 5 M NaOH, 2.2 mL of 1% ninhydrin, and 10 mL of water that thereafter were adjusted to 25 mL after inducing the reaction between metformin and ninhydrin. The reaction vessels subsequently were aliquoted to measure absorbance at 570 nm. To determine the maximum metformin concentration in the release media, the absorbance obtained from each vessel was compared to the reference absorbance obtained from a generated calibration.

### 2.7. In Vitro Release Kinetics

The release kinetics were computed using the data obtained from the drug release study, applying the Zero-order, First-order, Higuchi, and Korsmeyer-Peppas release models. The corresponding equations (Equations (4)–(7)) are as follows:

(4) $$Q_{t}=Q_{0}+k_{0}t,\;Zero\text{-}order$$

(5) $$\ln Q_{t}=\ln Q_{0}-k_{1}t,\;First\text{-}order$$

(6) $$Q_{t}=k_{H}t^{\frac{1}{2}},\;Higuchi$$

(7) $$\frac{Q_{t}}{Q_{\infty }}=kt^{n},\;Korsmeyer–Peppas$$

release models, respectively. In these equations, *Q* is the amount of drug released at time *t*, *Q*_0_ is the initial amount of drug, *Q_t_* is the total amount of drug released at infinite time, *k* is the release rate constant, and *n* is the release exponent indicative of the release mechanism. The model providing the best fit was selected based on the correlation coefficient (R2). These models are widely used for the analysis of drug release kinetics from pharmaceutical systems [25,26,27,28,29,30,31,32,33,34,35,36,37,38].

### 2.8. Cytotoxicity Studies

Cytotoxicity and cell viability experiments were performed using 3-(4,5-Dimethylthiazol-2-yl)-2,5-Diphenyltetrazolium Bromide (MTT) assay. OVCAR-3 cells (2 × 10^4^) were added into 96-well plates at a density of 3 × 10^3^ cells per well, respectively. After 24 h, different concentrations of metformin nanoparticles (150–200 µg/mL) were added to the cells. The wells without the nanoparticles were considered controls. A solution of MTT (2 mL) was added to each well at the end of 24, 48 and 72 h cycle. Optical density (OD) of each well was determined by absorbance at 570 nm wavelength using a microplate reader (Thermo-Scientific, Multiskan FC, Vantaa, Finland) for 4 h. The percentage of cytotoxicity value was determined by using the absorbance data, obtained from these experiments, and Equation (8).

(8) $$\%\;cytotoxicity=100-[\frac{O.D\;of\;eperimental\;value}{O.D\;of\;control\;value}]\times 100$$

The cytotoxic effect of the metformin nanoparticles drug sample on ovarian cancer OVCAR-3 cell lines was determined in real time with the xCelligence RT-SP device (ACEA Biosciences, San Diego, CA, USA).

### 2.9. In Vivo Antidiabetic Studies

#### 2.9.1. Induction of Diabetes

Type II Diabetes mellitus was induced in Male Wistar rats by subjecting them to fasting overnight before the administration of streptozotocin (50 mg/kg) dissolved in a citrate buffer (0.1 M, pH 4.5) intraperitoneally. The rats were assessed for diabetes by testing the fasting blood glucose (FBG) levels of the rats using a glucometer after 72 h. The rats with a FBG values above 250 mg/dL were used into the study.

#### 2.9.2. Treatment Groups

The experiment was performed by a previously reported method with some modifications [18,39]. Five (5) healthy male Wistar rats were randomly assigned to three (3) groups. Group I received 50 mg/kg of oral pure metformin daily, Group II received 50 mg/kg/day of metformin NPs, while Group III, which served as the control, received the vehicle alone. Blood samples were withdrawn from the tail vein of the Wistar rats on Days 0 (pre-treatment), 7, and 14. The glucose levels were read using a glucometer (Accu-Chek^®^, Roche, Basel, Switzerland) [18]. The efficacy of the antidiabetic agent was evaluated by monitoring blood glucose level fluctuations with time, and the results were recorded in mg/dL.

### 2.10. Data Analysis

All experiments were carried out in triplicate. Statistical analysis was performed using Microsoft Excel for data organization and SPSS version 29.0 for statistical testing. Differences in time-dependent metformin release between groups were evaluated using two-way ANOVA. A *p*-value less than 0.05 was considered statistically significant. Data are presented as mean ± standard deviation (*n* ≥ 3).

## 3. Results

### 3.1. SEM Analysis

**Figure 2:**
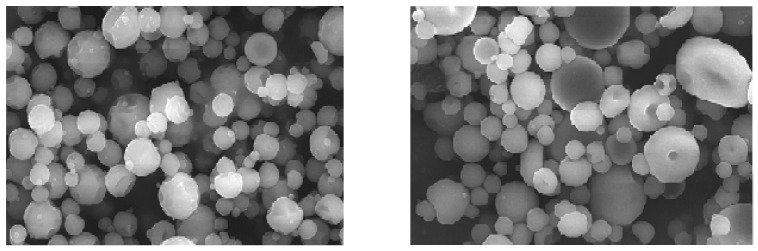
SEM images of optimized metformin nanoparticles (×10,000).

SEM images of metformin NPs showed separate and free-flowing nanoparticles with spherical and regular shape (Figure 2). The surface morphology of the nanoparticles was uniform and smooth, with no cracks, which may have contributed to the sustained release of the drug, as there was no leaching of metformin from the chitosan polymeric coat [21,23].

### 3.2. Zeta Potential, Particle Size, PDI, Entrapment Efficiency, and Loading Capacity of Metformin NPs

The data on average surface zeta potential, particle size, PDI, entrapment efficiency (EE%), entrapped drug amount, and loading capacity (LC%) of metformin-loaded NPs are summarized in Table 2. The mean zeta potential values of MN1–MN3 were highly positive, indicating strong electrostatic stabilization of the nanoparticles. Zeta potential is an important measure of colloidal dispersion stability. Its magnitude represents the degree of electrostatic repulsion between similarly charged particles in a dispersion [17,40,41]. A high zeta potential value enhances nanoparticle stability by preventing aggregation, whereas a low zeta potential may cause attractive forces to exceed repulsive forces, leading to flocculation of the colloidal dispersion [17]. The mean zeta potential values of batches MN1, MN2, and MN3 were +42.38 ± 0.02 mV, +45.29 ± 0.01 mV, and +49.06 ± 0.02 mV, respectively (Table 2), indicating that the formulated nanoparticles possessed a positive surface charge. This could be attributed to the use of chitosan, a biodegradable and positively charged polymer [17,23,42].

The average particle size of the formulated nanoparticles ranged from 74.28 ± 0.24 to 86.82 ± 0.31 nm. Also, the PDI values associated with the particle size measurements for batches MN1, MN2, and MN3 were 0.29 ± 0.02, 0.24 ± 0.01, and 0.32 ± 0.02, respectively, suggesting that the particles of the formulated nanoparticles were homogeneously dispersed. The polydispersity index (PDI) measures the heterogeneity of particle sizes within a sample. It is a critical parameter in nanoparticle formulation, as a lower PDI value indicates a more uniform particle size distribution. A PDI below 0.3 is desirable for pharmaceutical nanoparticles. A PDI value of 0 indicates that all particles are of the same size, while a value of 1 signifies a wide range of particle sizes [17,21,23]. The PDI values for batches MN1, MN2, and MN3 were 0.29 ± 0.02, 0.24 ± 0.01, and 0.32 ± 0.02, respectively, indicating homogeneous dispersion of the formulated nanoparticles.

The prepared nanoparticles showed metformin entrapment efficiency values between 68.42 ± 0.12% and 81.26 ± 0.26% that corresponded to entrapped drug amounts between 68.42 ± 0.12 mg and 162.52 ± 0.52 mg. Moreover, they showed metformin loading capacity values between 47.7 ± 1.42% and 51.7 ± 1.10%. Entrapment efficiency measures the ability of a nanocarrier to retain the drug, ensuring that a specific amount reaches the target site. It is expressed as the percentage of the drug successfully encapsulated within micelles or nanoparticles. The formulation of nanoparticles with varying polymer amounts results in different loading capacities and particle sizes. The formulated nanoparticles demonstrated entrapment efficiency values ranging from 68.42 ± 0.12% to 81.26 ± 0.26% (Table 2). The entrapment efficiency values reported were calculated using the indirect method based on supernatant analysis, while the direct method (nanoparticle digestion) was employed to confirm the accuracy of the obtained results. The results indicating metformin entrapment efficiency obtained by the direct method using Equation (2) closely matched those obtained by the indirect method using Equation (1), confirming the validity and accuracy of the initial entrapment efficiency measurements. Entrapped drug amounts varied from 68.42 ± 0.12 mg to 162.52 ± 0.52 mg, in line with the observed entrapment efficiencies, while the corresponding loading capacity values ranged between 47.7 ± 1.42% and 51.7 ± 1.10%. Moreover, an increase in polymer concentration led to a corresponding increase in both the mean particle size and the entrapment efficiency [21,40,42,43].

Hydrophilic drugs like metformin typically exhibit low entrapment efficiencies in some nanoparticle systems due to their rapid diffusion into the aqueous medium during nanoparticle formation [44,45]. However, the high entrapment efficiency observed in our study (up to 81.26%) may be attributed to several formulation-related factors. First, at the acidic pH used for chitosan solubilization, metformin’s biguanide groups are fully protonated, allowing strong electrostatic interactions with the negatively charged phosphate groups of TPP, which may have contributed to enhanced ionic complexation and retention of the drug within the forming nanoparticle matrix [19,42,46]. Second, the rapid ionic crosslinking that occurs upon dropwise addition of TPP under constant agitation likely led to near-instantaneous gelation and drug entrapment, minimizing the opportunity for drug diffusion out of the matrix [19,42,43]. Third, the relatively high drug-to-polymer ratio and increased viscosity of the chitosan solution in higher-polymer batches (e.g., MN3) may have slowed drug diffusion kinetics, improving drug retention [40,43,47]. These combined effects likely account for the enhanced entrapment of hydrophilic metformin in our chitosan nanoparticles.

### 3.3. FTIR Analysis

**Figure 3:**
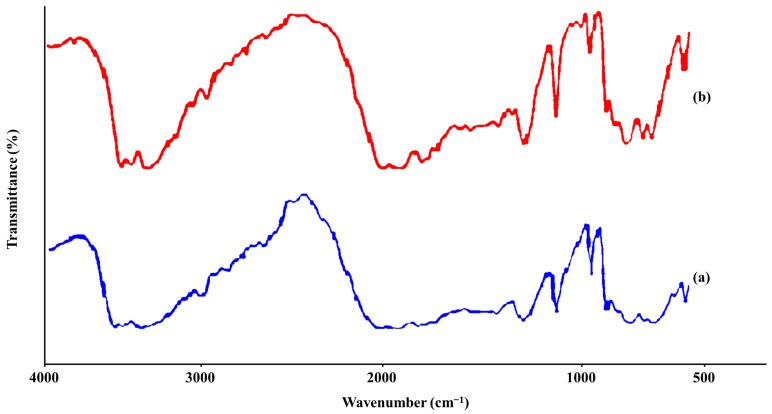
FTIR spectra of (**a**) pure metformin and (**b**) optimized (MN2) formulation.

The spectra showed characteristic peaks at 3366.59 cm^−1^ (H-N-H), 3289.85 cm^−1^ (N-H), 2542.84 cm^−1^ (C = N), 1621.73 cm^−1^ (C-N), 521.41 cm^−1^ (C-H), and the optimized nanoparticle formulation showed similar characteristic peaks (Figure 3). Metformin contains several functional groups, including N-H, C=N, H-N-H, C-H, and C-N [22]. The FTIR spectrum of pure metformin reveals peaks associated with these functional groups. The peaks observed were consistent with the metformin structure in terms of intensity and shape. A previous study by Sabbagh et al. [22] reported that the peaks at 3289.50 cm^−1^ and 3366.37 cm^−1^ correspond to the N-H functional group. The medium peak at 3150.48 cm^−1^ is attributed to the methyl group (C-H). The peaks at 1621.83 cm^−1^ and 1548.26 cm^−1^ are associated with the C=N functional groups, which are characteristic of biguanides. Peaks in the range of 1165.68–1038.79 cm^−1^ have been linked to the amine group (C-N). These peak assignments are based on a literature review of metformin vibrational spectra and the findings of previous studies conducted by Sabbagh et al. [22]. The FTIR spectrum of the optimized nanoparticles (NPs) shown in Figure 3 exhibited no significant differences compared to that of pure metformin, indicating that the active ingredient and the excipients used in the preparation were compatible.

### 3.4. XRD Analysis

X-ray diffraction (XRD) analysis is a method that provides extensive information on the crystallographic structure of a material [48,49]. It is based on the principle of monochromatic X-ray interference with crystal samples. XRD is a vital analytical technique used to analyze the crystal structure and arrangement of powdered samples. The intensity of the peaks is directly proportional to the crystal size: a longer peak strongly correlates with a larger crystal, and diffraction peaks with sharper intensities indicate greater crystallinity. Conversely, broader peaks suggest the presence of smaller crystals or an amorphous sample. The polymorphic nature of a drug is a crucial characteristic that determines its bioavailability and dissolution rate [48].

**Figure 4:**
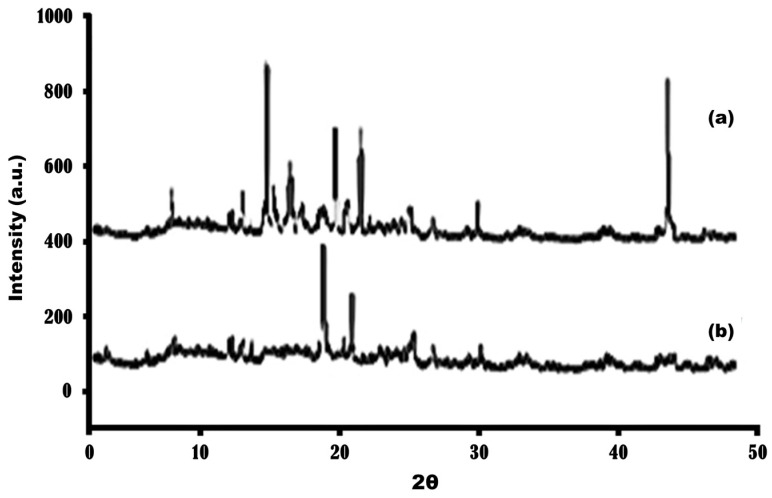
XRD spectra of (**a**) pure metformin and (**b**) optimized (MN2) formulation.

The XRD pattern of pure metformin (Figure 4a) exhibited multiple sharp and intense diffraction peaks at 2θ values of 14°, 19°, 22°, 30°, and 44°, confirming its highly crystalline nature. In contrast, the optimized nanoparticle formulation (MN2) (Figure 4b) showed a marked reduction in peak intensity, fewer distinguishable reflections, and noticeable peak broadening. Notably, the disappearance of the characteristic peak at 2θ ≈ 14° suggests disruption of the original crystal lattice and loss of long-range order, likely due to molecular dispersion of metformin within the chitosan–TPP matrix and interactions between the drug and polymer. Additionally, confinement within the nanoparticle structure may restrict crystal growth, contributing to reduced crystallite size and diminished diffraction coherence. While the diffraction peaks primarily arise from the metformin phase, the combined observations of peak disappearance, broadening, and reduced number of reflections indicate a transition from a crystalline to a partially amorphous state. Similar findings have been reported by Jagdale et al. [49]. In conclusion, these findings support successful encapsulation of metformin within the nanoparticle system, with partial amorphization likely due to drug–polymer interactions.

### 3.5. In Vitro Drug Release Studies

**Figure 5:**
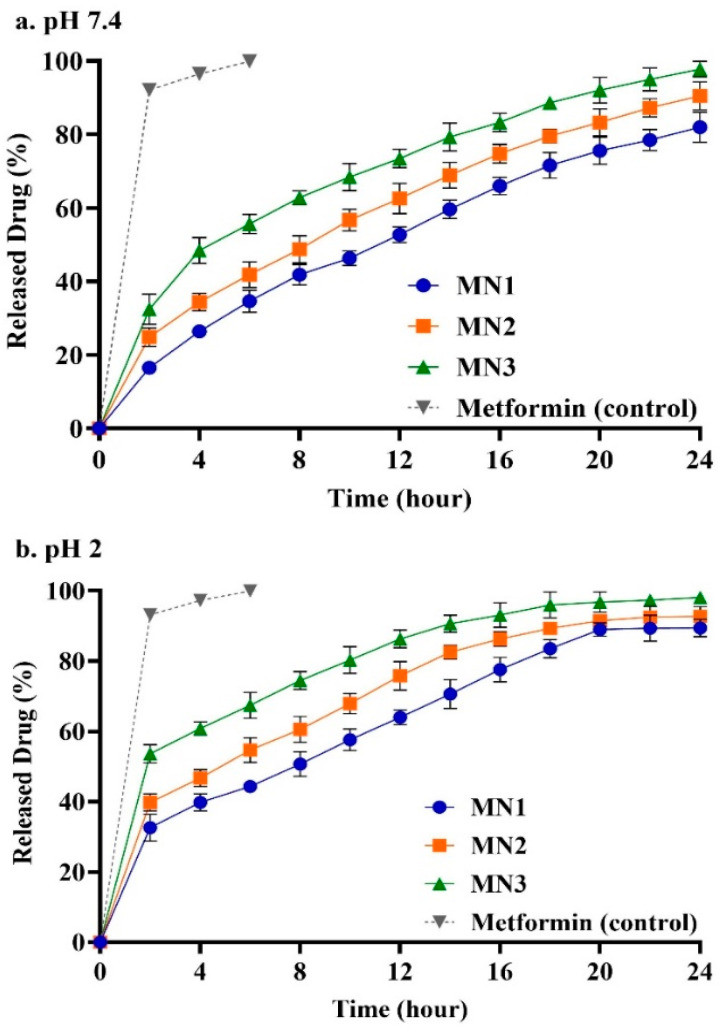
Drug release profiles of the formulated NPs MN1, MN2, and MN3 at (**a**) pH 7.4 and (**b**) pH 2.0. Data are presented as mean ± SD (*n* = 3). *p* < 0.05 was considered statistically significant.

The results of drug release at pH 7.4 (Figure 5a) showed that 16.54%, 24.82%, and 32.43% of metformin were released from batches MN1, MN2, and MN3, respectively, within the first 2 h, followed by sustained drug release for up to 24 h. At pH 2.0, as shown in Figure 5b, the nanoparticles showed similar amounts of released metformin to that achieved at pH 7.4 at the end of the release. However, the initial release was shown to occur at a faster rate in the release medium at pH 2.0, especially during the first 4 h. The initial rapid release from the metformin nanoparticles (NPs) could be attributed to the rapid dissolution of superficially adsorbed metformin, while the entrapped metformin within the polymeric NPs was released in a sustained manner. MN3 NPs exhibited the highest metformin release over 24 h, with a total release of 97.72% and 98.1% at pH 7.4 and pH 2.0, respectively [21,27]. Moreover, MN2 NPs achieved a total metformin release of 90.43% at pH 7.4 and 92.7% at pH 2.0, whereas MN1 NPs at pH 7.4 and pH 2.0 showed 81.92% and 89.4% average metformin release, respectively, over the release duration.

### 3.6. Released Metformin Determination

**Figure 6:**
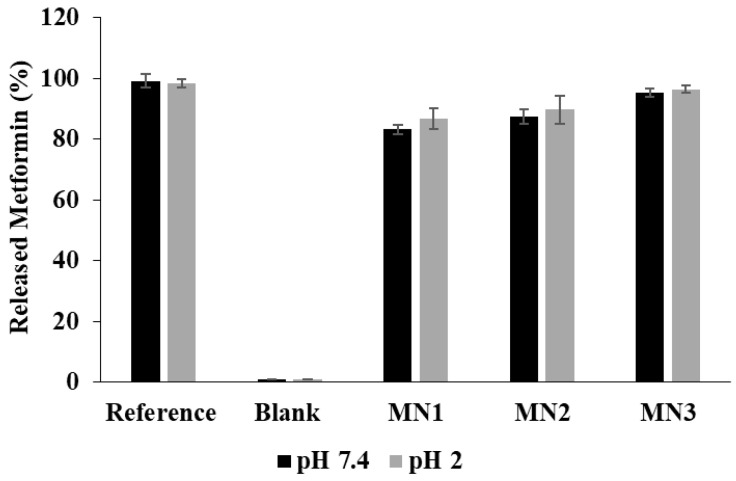
Peak metformin concentrations depicted in the release media as ninhydrin conjugate from MN1, MN2, and MN3 NPs after 24 h of drug release. Data are presented as mean ± SD (*n* = 3). *p* < 0.05 was considered statistically significant.

The maximum reached concentrations of metformin in the release media that were quantified at 232 nm subsequent to its release from MN1, MN2, and MN3 NPs were verified using a previously reported method [24]. Figure 6 shows the maximum metformin concentrations obtained from different metformin NPs as percentages in the release media. According to Figure 6, metformin release from MN3 NPs in pH 7.4 and pH 2.0 release media peaked at an average concentration of 95.98%, whereas MN1 and MN2 showed maximum average metformin concentrations of 85.06% and 88.56%, respectively, in the release media. The cumulative drug release from the metformin nanoparticle formulations was plotted as a function of time (Figure 5). In vitro release tests are widely used to predict in vivo drug absorption. This test is a crucial quality control measure for dosage forms and is currently employed to predict bioavailability. In some cases, it serves as an alternative to human bioequivalence studies, reducing the need for invasive experiments [50,51].

A strong correlation between the in vitro dissolution rate of most drugs and their in vivo bioavailability has been scientifically established. This concept, known as in vitro–in vivo correlation (IVIVC), suggests that in vitro dissolution studies can provide insights into how a drug will be absorbed when administered orally [50].

The in vitro drug release study demonstrated a sustained release profile for metformin-loaded chitosan NPs over 24 h, with variations in cumulative drug release depending on the formulation and pH conditions. The dialysis membrane method coupled with UV spectrophotometry at 232 nm revealed that MN3 exhibited the highest release, with 97.72% and 98.1% metformin release at pH 7.4 and pH 2, respectively. In comparison, MN2 and MN1 showed slightly lower cumulative release values of 90.43% and 81.92% at pH 7.4 and 92.7% and 89.4% at pH 2, respectively. Furthermore, statistical analysis using two-way ANOVA revealed no significant differences in metformin release across time points or in the interaction between time and formulation at either pH 7.4 or pH 2.0 (*p* > 0.05). However, marginally significant differences were observed between the formulations MN1, MN2, and MN3, with *p*-values of 0.0525 at pH 7.4 and 0.053 at pH 2, suggesting a consistent trend toward higher cumulative release from MN3 under both conditions.

The observed faster release rate of metformin at pH 2.0 compared to pH 7.4 can be attributed to several factors, primarily linked to the ionic gelation process and the pH-dependent interactions between the components of the delivery system. At pH 2.0, the increased concentration of H+ ions influences the ionic interactions between tripolyphosphate (TPP), chitosan, and metformin, leading to a faster drug release compared to the more neutral pH of 7.4 [52,53,54].

Chitosan, a cationic polysaccharide, forms ionic gel networks with anionic tripolyphosphate via electrostatic interactions between the amino groups of chitosan and the phosphate groups of TPP. This ionic gelation mechanism is crucial for the encapsulation and controlled release of metformin. At pH 7.4, where the environment is more neutral, these electrostatic interactions are stronger, and the ionic gel network is more stable, leading to slower drug release. In this pH environment, chitosan and tripolyphosphate maintain a tight network, limiting the rate at which metformin is released from the matrix.

Conversely, at pH 2.0, the environment becomes highly acidic due to the increased concentration of H+ ions. The lower pH disrupts the electrostatic interactions between chitosan and tripolyphosphate. The increased protonation of chitosan’s amino groups reduces its interaction with the anionic tripolyphosphate, weakening the ionic gel structure. This disruption leads to a faster release of metformin, as the drug is more easily displaced from the network. Similar pH-dependent release behavior has been reported in chitosan–polyphosphate nanoparticle systems [46], and acidification has also been shown to affect release from self-emulsifying or ionic-complex delivery systems [55]. Additionally, the increased protonation of metformin itself at lower pH enhances the solubility of the drug, facilitating its release from the matrix.

Furthermore, the ionic interactions between chitosan and metformin also play a role in the release dynamics. At pH 7.4, the ionization of metformin is less pronounced, which allows the drug to remain more strongly bound to the cationic amino groups of chitosan. At pH 2.0, the protonation of both chitosan and metformin may result in a less favorable ionic interaction, allowing metformin to be more easily displaced and released into the surrounding solution [52,53,54]. The combination of these factors—disruption of the chitosan–TPP ionic network, increased solubility of metformin, and weakened chitosan–metformin interactions—explains the faster release rate of metformin at pH 2.0 compared to pH 7.4.

These findings are consistent with previous studies that have demonstrated the pH-sensitive nature of chitosan-based drug delivery systems, where acidic environments lead to the dissociation of ionic networks and accelerate the release of encapsulated drugs [17,54,56,57]. Additionally, the protonation of both the chitosan polymer and the drug itself in acidic environments has been shown to enhance the release rate by reducing electrostatic binding and increasing drug solubility.

In this study, pH 2.0 was selected as the acidic release medium to simulate the gastric environment, although the pharmacopeial standard for simulated gastric fluid is pH 1.2. This choice was intentional to preserve the structural integrity of the chitosan–TPP nanoparticles during the in vitro release period. Chitosan is a cationic polymer that is highly protonated and soluble under strongly acidic conditions (typically below pH 4) [17,52]. At pH 1.2, ionically crosslinked chitosan–TPP nanoparticles are prone to excessive swelling, surface erosion, or even disintegration, which can result in burst release artifacts or loss of formulation fidelity [54,57,58]. By using pH 2.0 as a moderately acidic environment, the release study maintained physiologically relevant conditions while minimizing nanoparticle destabilization. This approach is consistent with previous studies that adopted pH 2.0 to assess gastric-phase drug release from acid-sensitive chitosan systems [17,54].

As previously used for entrapment efficiency verification, the ninhydrin assay was also applied here to confirm drug quantification, ensuring consistency and reliability across all analytical assessments. To confirm the results obtained from the UV spectrophotometric method, a secondary quantification method utilizing ninhydrin-based spectrophotometric analysis at 570 nm was employed at the end of 24 h. This method is based on the reaction between metformin and ninhydrin under alkaline conditions, producing a spectrophotometrically detectable violet-colored complex [24]. The results obtained from this method (Figure 6) showed maximum metformin concentrations of 95.98% for MN3, 88.56% for MN2, and 85.06% for MN1, which closely correlate with the values obtained from the UV spectrophotometric release study.

Comparing the data from both methods, the minor variations observed can be attributed to differences in detection wavelengths and the sensitivity of the respective techniques. The UV spectrophotometric method at 232 nm primarily detects metformin’s native absorbance, while the ninhydrin-based method provides an indirect quantification through a chemical reaction, which could introduce slight variations in measurement precision. However, the strong correlation between the two methods supports the accuracy of the drug release results, confirming that metformin release from the nanoparticles follows the expected diffusion-controlled mechanism as indicated by the Higuchi model [28], as will be discussed later.

The confirmation of the release data through an independent analytical technique further validates the reliability of the dialysis membrane-based UV spectrophotometric method for assessing metformin release from nanoparticles. The findings reaffirm that MN3 exhibits the highest sustained release, followed by MN2 and MN1, consistent with differences in polymer composition and drug entrapment efficiency. This study highlights the robustness of the applied release methods and supports the potential use of chitosan-based nanoparticles for controlled metformin delivery, potentially improving dose consistency and patient adherence. Similar in vitro sustained-release and cellular-uptake evaluations are commonly used in nanodelivery development [59].

### 3.7. Results of the In Vitro Release Kinetics

The results of drug release kinetics at pH 7.4 (Table 3) and pH 2 (Table 4) revealed that the drug release kinetics from the NPs simulated first-order and Higuchi release kinetics [28,29]. MN1 followed first-order release kinetics (r^2^ = 0.988) at pH 7.4, whereas it followed the Higuchi model (r^2^ = 0.989) at pH 2. These two r^2^ values indicated concentration-dependent and diffusion-controlled drug release mechanisms, respectively. MN2 and MN3 followed the Higuchi model at pH 7.4 (r^2^ = 0.991 and 0.990, respectively), confirming diffusion-controlled release mechanisms for these formulations. Comparatively, MN1 and MN2 at pH 2 exhibited a slight shift from the Higuchi model to first-order release kinetics with r^2^ values of 0.986 and 0.989, respectively. The release kinetics at pH 7.4 and pH 2 of MN1, MN2, and MN3 were analyzed using four different mathematical models: zero-order, first-order, Higuchi, and Korsmeyer–Peppas [25,26,27,28,29,30,31,32,33,34,35,36,37,38]. The best-fitting model was determined based on the coefficient of determination (r^2^) values, which indicate how well each model describes the experimental data [29].

For MN1, the highest r^2^ value (0.988) was observed for the first-order model, closely followed by the Higuchi model (r^2^ = 0.987). This suggests that drug release from MN1 follows concentration-dependent kinetics, where the release rate decreases over time as the drug concentration in the formulation decreases [29]. The high r^2^ value for the Higuchi model indicates that diffusion also plays a role in the release mechanism [28]. However, the stronger fit of the first-order model suggests that the release is not purely diffusion-driven but rather influenced by the concentration of the remaining drug in the matrix. The zero-order model (r^2^ = 0.971) exhibited slightly lower correlation, indicating that the release is not at a constant rate. The Korsmeyer–Peppas model showed an ɳ value of 0.539, indicating non-Fickian diffusion, suggesting that both diffusion and polymer relaxation/swelling contribute to the release mechanism [30,31,32,33,34].

At pH 2, MN1 also exhibited high correlation with the First-order model (r^2^ = 0.971) and the Higuchi model (r^2^ = 0.989). However, the ɳ value (0.341) was lower than that observed at pH 7.4, indicating a greater contribution of Fickian diffusion and suggesting a predominantly diffusion-controlled release mechanism.

For MN2, the highest r^2^ value (0.991) was observed for the Higuchi model, indicating that the drug release primarily follows a diffusion-controlled process [28]. The first-order model also provided a relatively good fit (r^2^ = 0.964), but the slightly lower value suggests that diffusion is the dominant release mechanism rather than concentration-dependent dissolution. This means that the drug moves through the formulation matrix by passive diffusion, consistent with porous or hydrophilic matrix systems [27,30,31,32,34,35]. The zero-order model (r^2^ = 0.950) exhibited lower correlation, reinforcing that drug release is not occurring at a constant rate. The Korsmeyer–Peppas model showed an ɳ value of 0.622, indicating anomalous (non-Fickian) transport, meaning that both diffusion and polymer relaxation contribute to the release process [30,31,32,33,34].

Additionally, at pH 2, MN2 followed a similar pattern, with the Higuchi model providing a strong correlation (r^2^ = 0.978), followed by the first-order model (r^2^ = 0.986). However, the ɳ value was lower (0.283), suggesting a shift toward a more Fickian diffusion-controlled release mechanism. The zero-order model showed a lower correlation (r^2^ = 0.841), indicating that a constant release rate is not maintained [29,31,33,35].

For MN3, the Higuchi model showed the best fit (r^2^ = 0.990), followed by the first-order model (r^2^ = 0.968). This pattern aligns with MN2, reinforcing the idea that diffusion is the dominant release mechanism in MN3 as well [28]. The zero-order model showed the lowest correlation (r^2^ = 0.873), suggesting that release is not occurring at a constant rate. The ɳ value in the Korsmeyer–Peppas model was 0.713, indicating an anomalous transport mechanism where both diffusion and polymer relaxation play a role [30,31,32,33,34]. The increasing ɳ value from MN1 to MN3 suggests that MN3 exhibits more erosion-controlled release, possibly due to increased polymer hydration or degradation [35,38,60].

Furthermore, at pH 2, MN3 followed a similar trend, with the Higuchi model showing a strong correlation (r^2^ = 0.921), but the first-order model had a slightly higher r^2^ value (0.989), suggesting a stronger concentration-dependent release behavior in this case. Interestingly, the Korsmeyer–Peppas model did not provide an ɳ value, which may indicate deviation from the model assumptions, possibly due to a more complex release mechanism [36,37].

The first-order model provided the best fit for MN1, while the Higuchi model best described the release behavior for MN2 and MN3, indicating that diffusion is the primary mechanism for MN2 and MN3, whereas MN1 is more influenced by drug concentration [28,29]. The increasing ɳ values from MN1 (0.539) to MN3 (0.713) suggest a shift from primarily diffusion-controlled release in MN1 toward a more erosion- or swelling-controlled release in MN3. The differences in release kinetics indicate that MN3 has the highest release rate, likely due to increased polymer porosity or degradation, whereas MN1 provides a more controlled, sustained release profile [27,34,35,38].

The release kinetics of MN1, MN2, and MN3 suggest a transition from a more controlled and sustained release mechanism in MN1 to a faster, diffusion-dominated release in MN3. The strong fit of the Higuchi model for MN2 and MN3 suggests a dominant diffusion mechanism, while the first-order model fit for MN1 indicates concentration-dependent release. Similar findings were reported by Öztürk et al. [26], who prepared dexketoprofen-loaded Eudragit RL 100 NPs and found that the Korsmeyer–Peppas model was suitable for describing drug release from their formulated polymeric NPs. The increasing ɳ values in the Korsmeyer–Peppas model suggest a shift toward an erosion-based mechanism for MN3. These findings are essential for optimizing drug formulations, balancing controlled release with the desired therapeutic effect [38].

### 3.8. Results of the Cytotoxicity Assay

The cytotoxicity evaluation of metformin NPs demonstrated a clear dose- and time-dependent antiproliferative effect against OVCAR-3 ovarian cancer cells. The statistical analysis further substantiated that these effects were not only progressive but also significantly different between treatment conditions, indicating a structured and quantifiable biological response.

**Figure 7:**
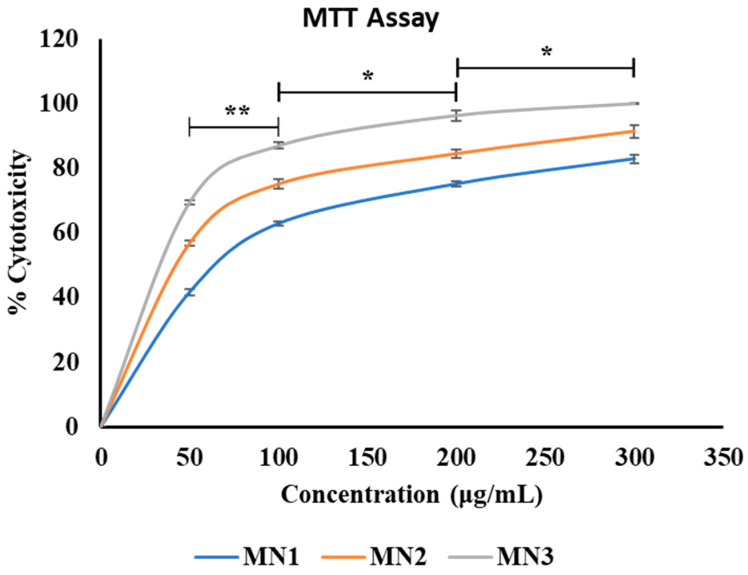
Graph showing the cytotoxic effect of metformin nanoparticles on the viability of OVCAR3 cancer cells with varying concentrations. Data are presented as mean ± SD (*n* = 3). * *p* < 0.05 and ** *p* < 0.01.

**Figure 8:**
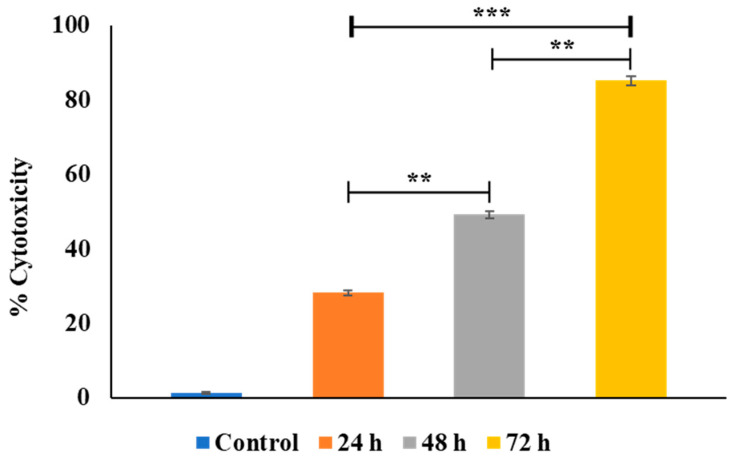
Cytotoxic effects of the metformin nanoparticles on ovarian cancer (OVCAR3) cell lines after 24, 48, and 72 h. Data are presented as mean ± SD (*n* = 3). ** *p* < 0.05 and *** *p* < 0.001 were considered statistically significant.

In the concentration-dependent assessment (Figure 7), cytotoxicity increased significantly with increasing nanoparticle concentration. Pairwise comparisons revealed that the increase from 50 to 100 µg/mL was highly significant (*p* < 0.01), while further increases from 100 to 200 µg/mL and from 200 to 300 µg/mL remained statistically significant (*p* < 0.05). This pattern suggests that the largest incremental cytotoxic effect occurs at lower-to-mid concentration ranges, after which the response begins to plateau. Such behavior is consistent with dose–response saturation kinetics, where increasing concentrations produce diminishing incremental effects once a substantial proportion of cells are already compromised. The calculated IC50 values further support this concentration-dependent behavior, demonstrating a progressive reduction with increasing exposure duration. The IC50 decreased from 95.24 µg/mL at 24 h to 48.72 µg/mL at 48 h and further to 25.81 µg/mL at 72 h, indicating that lower concentrations of metformin nanoparticles are required to achieve 50% cytotoxicity over time. This shift reflects an increase in apparent potency and is consistent with the observed plateauing effect at higher concentrations, where maximal cytotoxicity is approached. Similarly, the time-dependent cytotoxicity profile (Figure 8) showed a significant and progressive increase in cytotoxic effect with prolonged exposure. The transition from 24 to 48 h and from 48 to 72 h exhibited statistically significant differences (*p* < 0.01), while the overall increase from 24 to 72 h was highly significant (*p* < 0.001). These findings indicate that prolonged exposure enhances nanoparticle-mediated cytotoxicity, likely due to sustained intracellular accumulation and extended interaction with cellular targets. The combined dose- and time-dependent trends, together with the decreasing IC50 values, suggest that metformin nanoparticles exert their cytotoxic effects through a cumulative mechanism, where both increased concentration and extended exposure contribute to enhanced efficacy. The reduction in IC50 over time further implies that exposure duration becomes a critical determinant of cytotoxic potency, potentially reflecting time-dependent intracellular drug release and retention. The consistent non-cytotoxic effect of the control group over time validated the cytotoxicity assay, which affirms that apoptosis seen in the treated groups was genuinely due to the effect of the drug-loaded nanoparticles (Figure 8). A previous study using SKOV3 human ovarian carcinoma cells also reported enhanced anti-proliferative and pro-apoptotic effects of metformin-loaded PLGA–PEG nanoparticles compared with free metformin [61]. Overall, the statistically validated differences between treatment groups confirm that metformin NPs exhibit a controlled, progressive, and significantly enhanced cytotoxic profile, supporting their potential as an effective nanocarrier system for anticancer therapy.

### 3.9. Results of the Antidiabetic Studies

The results of the antidiabetic efficacy of metformin NPs in monitoring fasting blood glucose (FBG) levels in streptozotocin-induced diabetic rats were evaluated over a 14-day treatment period (Table 5). The Wistar rats in Group II (metformin NPs) experienced a steady and significant decrease in FBG levels, reducing from 286.52 ± 1.20 mg/dL on Day 0 to 118.42 ± 0.45 mg/dL on Day 14 (*p* < 0.05 vs. Day 0; † *p* < 0.05 vs. control). Similarly, FBG levels in Group I (pure metformin) reduced by 36.61%, decreasing from 281.41 ± 1.28 mg/dL to 178.41 ± 0.82 mg/dL (*p* < 0.05 vs. Day 0). In contrast, consistently high blood glucose levels were seen in the control group (Group III). The increased bioavailability of the nanoparticles resulting from the nanoscale particle size, increased surface area, and sustained-release effect may be responsible for the significantly higher hypoglycaemic effect observed with metformin nanoparticles (*p* < 0.05). Similar results were reported for metformin-loaded nanoparticles by Kumar et al. and Khatri et al. [18,39].

## 4. Conclusions

This study successfully formulated and characterized metformin-loaded nanoparticles (NPs) using chitosan as a biodegradable polymer. FTIR analysis confirmed the compatibility of metformin with the excipients, while XRD analysis indicated a reduction in crystallinity, suggesting successful encapsulation. The nanoparticles exhibited a smooth surface morphology, uniform dispersion (PDI ≤ 0.3), and high zeta potential (+42.38 to +49.06 mV), ensuring stability. The entrapment efficiency ranged from 68.42% to 81.26%, with particle sizes between 74.28 ± 0.24 and 86.82 ± 0.31 nm. In vitro drug release studies demonstrated an initial burst release followed by sustained release over 24 h, with formulation MN3 achieving the highest release at 97.72% and 98.1% at pH 7.4 and pH 2.0, respectively. All metformin NPs exhibited a faster release after decreasing the pH of the release medium from 7.4 to 2.0. In the majority of the studied drug release profiles, the release kinetics followed the Higuchi model, indicating diffusion-controlled drug release. The metformin nanoparticles demonstrated a potent dose-related and time-dependent cytotoxic effect against ovarian cancer cell lines and in vivo blood glucose lowering effect compared to the conventional dosage forms and control (*p* < 0.05). These findings suggest that the formulated nanoparticles can enhance the sustained release of metformin, potentially improving its therapeutic efficacy and patient compliance.

## Figures and Tables

**Table 1:** Formula of metformin nanoparticles.

Batches	Chitosan (mg)	TPP (mg)	Metformin (mg)
MN1	50	20	100
MN2	100	30	150
MN3	150	40	200

**Table 2:** Characterization of metformin-loaded NPs, including surface zeta potential, particle size, PDI, entrapment efficiency (EE%), entrapped drug (ED) amount, and loading capacity (LC%).

Batches	Zeta Potential (mV)	Particle Size (nm)	PDI	EE (%)	ED (mg)	LC (%)
MN1	+42.38 ± 0.14	74.28 ± 0.24	0.29 ± 0.02	68.42 ± 0.12	68.42 ± 0.12	51.7 ± 1.10
MN2	+45.29 ± 0.11	81.46 ± 0.17	0.24 ± 0.01	76.84 ± 0.11	115.26 ± 0.17	48.8 ± 0.99
MN3	+49.06 ± 0.12	86.82 ± 0.31	0.32 ± 0.02	81.26 ± 0.26	162.52 ± 0.52	47.7 ± 1.42

**Table 3:** Drug release kinetics of metformin NPs using the release medium at pH 7.4.

Models	Zero		First		Higuchi		Korsmeyer and Peppas	
Formulations	r^2^	K_0_	r^2^	K_1_	r^2^	K_H_	r^2^	ɳ
MN1	0.971	3.351	0.988	−0.031	0.987	17.719	0.817	0.539
MN2	0.950	3.459	0.964	−0.043	0.991	19.238	0.763	0.622
MN3	0.873	3.421	0.968	−0.054	0.990	19.547	0.701	0.713

**Table 4:** Drug release kinetics of metformin NPs using the release medium at pH 2.0.

Models	Zero		First		Higuchi		Korsmeyer and Peppas	
Formulations	r^2^	K_0_	r^2^	K_1_	r^2^	K_H_	r^2^	ɳ
MN1	0.917	4.561	0.971	0.096	0.989	19.002	0.976	0.341
MN2	0.841	4.960	0.986	0.111	0.978	20.906	0.979	0.283
MN3	0.719	5.423	0.989	0.158	0.921	23.161	0.000	None

**Table 5:** Results of the FBG levels in the various treatment groups.

Group	Day 0 (mg/dL)	Day 7 (mg/dL)	Day 14 (mg/dL)
I	281.41 ± 1.28	228.29 ± 0.49	178.41 ± 0.82 *
II	286.52 ± 1.20	201.48 ± 1.57	118.42 ± 0.45 *†
III	274.21 ± 1.21	281.57 ± 0.82	289.52 ± 0.63

Key: * indicates significance vs. Day 0 within the same group (*p* < 0.05), whereas † indicates significant vs. control at the same timepoint (*p* < 0.05).

## Data Availability

All relevant data are within the manuscript.
